# Oxidative stress in multiple sclerosis—Emerging imaging techniques

**DOI:** 10.3389/fneur.2022.1025659

**Published:** 2023-01-12

**Authors:** Christopher Hollen, Lee E. Neilson, Ramon F. Barajas, Ian Greenhouse, Rebecca I. Spain

**Affiliations:** ^1^Department of Neurology, Veterans Affairs Medical Center, Portland, OR, United States; ^2^Department of Neurology, Oregon Health and Sciences University, Portland, OR, United States; ^3^Department of Radiology, Neuroradiology Section, Oregon Health & Sciences University, Portland, OR, United States; ^4^Advanced Imaging Research Center, Oregon Health & Science University, Portland, OR, United States; ^5^Knight Cancer Institute, Oregon Health & Science University, Portland, OR, United States; ^6^Department of Human Physiology, University of Oregon, Eugene, OR, United States

**Keywords:** progressive multiple sclerosis, oxidative stress, magnetic resonance spectroscopy, glutathione, positron emission tomography, magnetic resonance imaging

## Abstract

While conventional magnetic resonance imaging (MRI) is central to the evaluation of patients with multiple sclerosis, its role in detecting the pathophysiology underlying neurodegeneration is more limited. One of the common outcome measures for progressive multiple sclerosis trials, atrophy on brain MRI, is non-specific and reflects end-stage changes after considerable neurodegeneration has occurred. Identifying biomarkers that identify processes underlying neurodegeneration before it is irreversible and that reflect relevant neurodegenerative pathophysiology is an area of significant need. Accumulating evidence suggests that oxidative stress plays a major role in the pathogenesis of multiple neurodegenerative diseases, including multiple sclerosis. Imaging markers related to inflammation, myelination, and neuronal integrity have been areas of advancement in recent years but oxidative stress has remained an area of unrealized potential. In this article we will begin by reviewing the role of oxidative stress in the pathogenesis of multiple sclerosis. Chronic inflammation appears to be directly related to the increased production of reactive oxygen species and the effects of subsequent oxidative stress appear to be amplified by aging and accumulating disease. We will then discuss techniques in development used in the assessment of MS as well as other models of neurodegenerative disease in which oxidative stress is implicated. Multiple blood and CSF markers of oxidative stress have been evaluated in subjects with MS, but non-invasive imaging offers major upside in that it provides real-time assessment within the brain.

## Introduction

Multiple sclerosis (MS) is the most common inflammatory neurologic disease impacting young adults worldwide. The overall annual economic burden of MS in the United States is approximately $85 billion, placing it among the costliest of chronic diseases (1). In the majority of cases, MS begins with a relapsing-remitting (RRMS) course with unpredictable demyelinating events affecting the brain, optic nerves and spinal cord causing episodes of visual disturbance, weakness, sensory loss, coordination problems, bladder and bowel dysfunction, mood and cognitive changes, and other symptoms. Incomplete recovery from relapses leads to accumulated disability which leads to physical dependence, immobility, and inability to maintain employment (2, 3). Most RRMS patients go on to develop progressive clinical worsening in the absence of relapses about 20 years after initial disease onset, termed secondary progressive MS (SPMS) (4). Roughly 15% of MS cases begin with a progressive disease course from onset, termed primary progressive MS (PPMS) (5). RRMS is characterized broadly by an inflammatory process whereas PPMS and SPMS are considered to be driven predominantly by a neurodegenerative process.

Conventional magnetic resonance imaging (MRI) is standard of care for the evaluation of patients with relapsing forms of MS with exquisite sensitivity to changes in lesion burden. Standard T1- and T2-weighted MRI findings are biologically nonspecific and temporally dependent, but, reflect the underlying neuroinflammatory process associated demyelination. None the less, imaging features are all clinically informative and correlate to outcome measures, such as disease disability, prognosis, and response to MS neuroinflammatory disease-modifying therapy (6). Despite the clinical utility of MRI features, they often fail to prospectively capture the sequela of a chronic neuroinflammatory process eventually leading to neurodegeneration changes in both relapsing and progressive forms of MS. Global and regional brain atrophy can be detected early in MS and becomes more prominent in progressive phases of the disease. Thus brain atrophy, while a nonspecific and late biomarker of disease status, has become the most broadly used imaging marker for MS neurodegeneration in progressive MS clinical trials (7).

Identifying biomarkers of neurodegeneration before it becomes irreversible and that reflect relevant MS pathophysiology is an area of unmet need. Indicators of oxidative stress (OS) are promising candidates as a means of assessing a critical aspect of the neurodegenerative process in MS because OS likely occurs early and widely in the cascade leading to the degradation of neurons. In this review, we discuss the role of OS in MS pathophysiology and present imaging techniques with potential for demonstrating OS *in vivo* in MS.

## Oxidative stress in neurodegenerative disease

Fundamentally, OS is generated by an imbalance of reduction and oxidation (redox) reactions wherein highly reactive free radicals seek electrons from other molecules, which then become free radicals themselves, causing a cascade that leads to cellular damage. Agents that induce OS include reactive oxygen species (ROS) such as superoxide radicals and hydrogen peroxide and reactive nitrogen species (RNS) such as nitric oxide radicals and peroxynitrite. Mitochondria are the biggest generators of ROS through oxidative respiration. ROS and RNS in the CNS are also directly produced by microglia and macrophages with the expression of enzymes such as NADPH-oxidases, myeloperoxidase, nitric oxide synthase. Thus, the brain, with its high consumption of oxygen and lipid-rich content is especially susceptible to OS. OS occurs with aging and in multiple neurodegenerative disorders of the CNS in addition to MS including Alzheimer's disease (8, 9), Parkinson disease (10), ALS (11), and Huntington disease (12).

The CNS hosts multiple defenses against OS, which are divided into the antioxidant enzyme system and non-enzymatic low-molecular-weight antioxidants (13). The enzyme defense system includes superoxide dismutases (SODs), glutathione peroxidase, glutathione reductase, and catalase. The non-enzymatic antioxidants most relevant to the CNS include glutathione, ascorbic acid, and melatonin. Both of these defense systems are controlled by the transcription factor nuclear factor (erythroid-derived 2)-like 2 (Nrf2). When activated, Nrf2 increases the expression of multiple antioxidants, enzymes, and transporters (14). Under normal conditions, anti-oxidant defense mechanisms mediated by the Nrf2 system and the most abundant anti-oxidant in the CNS, glutathione (GSH), prevent much of the cerebral injury that might occur. However, when these defense mechanisms are outstripped, cellular damage and potential neurodegeneration occurs (15). Once this neurodegeneration surpasses an individual's functional reserve, clinical decline becomes apparent (16).

## Oxidative stress in MS

While clinical phenotypes of MS may be distinct, on histopathological investigation there may be a spectrum of pathologies even within a single person. Hallmarks of the early stages of relapsing MS include focal inflammatory plaques characterized by an influx of T cells across the blood brain barrier causing subsequent demyelination, axonal loss, and reactive gliosis. In the later stages of SPMS and in PPMS from onset, microglial activation is diffusely present throughout the brain as well as at lesion edges (smoldering lesions) and meningeal B-cell follicles are characteristic leading to diffuse atrophy (17). Additional mechanisms contributing to MS neurodegeneration include cortical demyelination, meningeal lymphoid follicles, and diffuse microglial activation which are the core histologic features of progressive MS (18–20). In many cases these pathologies do not exist discretely; rather simultaneous inflammation and neurodegeneration may be present, blurring the clinical distinctions between relapsing and progressive MS (21). Chronic inflammation appears to play a role in initiating the neurodegenerative process characteristic of PMS (22–24). Whether inflammation and neurodegeneration are primary or secondary processes and how they might interact is controversial (25, 26).

In the context of MS, oxidative injury is demonstrated by histopathology throughout the duration of the disease with evidence of OS found in actively enhancing lesions from relapsing MS lesions as well as non-enhancing (non-active) lesions in progressive MS (27, 28). Within MS lesions, enzymes related to free radical production are increased (27). Oxygen free radicals are generated directly by inflammation, particularly due to microglia. This innate immune system activation is present in the relapsing stage but may also be present in the progressive phases of MS, though blood-brain-barrier damage may be beneath the sensitivity of gadolinium-enhanced MRI (23).

Mitochondrial dysfunction is present in MS and along with causing axonal dysfunction produces excessive ROS (23, 29). In fact, exposing healthy rat neurons to CSF from patients with progressive MS induced mitochondrial dysfunction and axonal damage, and caused bioenergetic failure. Importantly, this study also identified the role of ceramide as the mediator of this mitochondrial failure (30). Short-chain ceramides have been shown to induce mitochondrial alterations such as ROS production and permeabilization of the mitochondrial membrane leading to neuronal death (31). A functional screening study that performed live-imaging of rat neurons treated with the CSF of progressive MS patients demonstrated a morphological change (elongation) of mitochondria with decreased activity of complexes I, III, IV that correlated with axonal damage.

Iron concentration within the parenchyma increases with aging but is more pronounced in the context of progressive MS. Based on *in vitro* studies, iron appears to accentuate ROS release by NADPH oxidase in microglia (32). The presence of divalent iron ions generated by oligodendrocytes damaged by MS during demyelination may amplify free radical formation (33). Iron is primarily stored in oligodendrocytes, and high intracytoplasmic accumulation of iron in oligodendrocytes may explain preferential susceptibility of these cells to degeneration in response to OS (23, 34). Excess iron in the extracellular space, a consequence of oligodendrocyte destruction, may be taken up by activated microglia which alters them to become dystrophic and degenerative, creating a cyclical process causing further oxidative damage. Since iron increases in the brain normally with age, this damaging cycle is more common in those with secondary progressive MS (23). While iron does play a role in OS, many regions accumulate iron that do not exhibit excessive free radical generation. This makes iron-sensitive techniques such as susceptibility weighted imaging, insufficient biomarkers for assessing oxidative stress.

While histopathological evidence demonstrates the presence of OS, laboratory biomarkers of OS have been limited by the inability to directly access the CNS. However, a recent meta-analysis indicated increased blood and CSF malondialdehyde and decreased blood albumin levels which strongly suggests a link between OS and MS (35). Additionally, CSF and urinary 8-iso-PGF2α levels, which reflect the degree of lipid peroxidation due to ongoing oxidative stress, were found to be significantly higher in MS subjects than in controls (36, 37). GSH and redox values of GSH/GSSH may be measured in both peripheral blood and CSF. Challenges to measuring glutathione include that the quantity of intracerebral GSH is much higher intracellularly (10 ×) than extracellularly, and the resulting CSF concentrations are so low in CSF as to be difficult to detect and may not accurately reflect intracerebral GSH concentration (38). Studies of erythrocyte glutathione levels as well as glutathione peroxidase activity have suggested decreases of this critical antioxidant in MS (38). While taken as a whole these lab markers do suggest cellular redox pathway changes in MS, due to the heterogeneity of techniques, imaging markers may offer a superior means of real-time *in vivo* quantification and provide more anatomically precise assessments of OS-related pathology.

## Imaging oxidative stress

Development of OS imaging techniques has emerged in the field of neurodegenerative diseases due to altered mitochondrial oxidative metabolism (39–41). More recently, imaging with magnetic resonance imaging (MRI), magnetic resonance spectroscopy (MRS), and positron emission tomography (PET) may allow clinically-relevant quantification of OS non-invasively *in vivo*. These techniques have varied approaches in assessing OS pathophysiology. Some directly interrogate for the presence of ROS, while others target downstream consequences or related processes. A summary of these imaging modalities, targets, and key studies are presented in Table 1. Some of the specific advantages and disadvantages of these techniques are identified in Table 2. While few of these techniques have been studied in MS, these techniques do aim to target OS pathways relevant to MS. There is significant interest in OS imaging as a potential means of developing earlier, more effective biomarkers of neurodegeneration. While histopathological evidence strongly points to the role of OS in MS neurodegeneration, assessments of OS throughout the course of MS *in vivo* alongside assessments of downstream neurodegenerative pathophysiology would provide critical information regarding the link between OS and neurodegeneration. Finding techniques that assess localized OS within the brain prior to colocalized degeneration would be an important step in this direction. Such a technique should be able to be feasibly applied to demonstrate efficacy of interventions in preventing both OS and the development of neurodegeneration. This will require sensitive, high-resolution imaging techniques performed alongside established neurodegenerative biomarkers.

**Table 1:** OS imaging techniques used in MS and other neurodegenerative disease models *in vivo*.

**Imaging modality**	**Target**	**Studies of interest**	**Technique**	**Human subjects?**	**Study population/model**	**Findings**
MRI	Endogenous paramagnetic free radicals	Berkowitz et al. (42)	Quench-Assisted (QUEST) MRI assessing R1 at 7T	No	Mouse model of Alzheimer disease and Angelman syndrome	Alzheimer-like mouse models showed an abnormal gradient of free radical production along the CA1 dorsal–ventral axis. Angelman syndrome models showed elevated free radical levels identified in dorsal and ventral CA1
		Berkowitz et al. (43)	Quench-Assisted (QUEST) MRI assessing R1 at 7T	No	Mouse model of aging and repeated anesthesia	Age- and isoflurane-related oxidative stress localized to the stratum lacunosum of CA1
	Mito-TEMPO	Zhelev et al. (44)	MRI with mito-TEMPO contrast	No	MPTP-treated mice	High oxidative activity in dopaminergic tissues of MPTP-treated mice
	Proton exchange rate	Ye et al. (45)	Single slice chemical exchange saturation transfer (CEST) MRI at 3T	Yes	RRMS	For gadolinium negative lesions, *k*_ex_ appears to differentiate slowly expanding lesions with chronic inflammation and potential OS
	Nitroxyl radicals	Yamato et al. (46)	Overhauser-enhanced MRI	No	Rat model of Parkinson disease (6-OHDA lesions)	Mitochondrial dysfunction in the lesioned hemisphere was reflected in the OMRI image and was associated with decreased dopamine levels
MRS	Glutathione (GSH)	Srinivasan et al. (47)	MRSI at 7T using band selective inversion with gradient dephasing (BASING)	Yes	MS and healthy controls (HC)	Reduced GSH in gray matter and white matter lesions in MS compared to controls
		Choi et al. (48)	Selective multiple quantum chemical shift imaging (CSI) at 3T	Yes	SPMS and HC	Reduced GSH in SPMS compared to controls, most notable in the frontal regions
		Choi et al. (49)	Selective multiple quantum CSI at 3T	Yes	RRMS, PPMS, SPMS, HC	Reduced GSH in MS compared to controls, GSH lower in progressive MS compared with RRMS
	Vitamin C/dehydroascorbate (DHA)	Qin et al. (50)	Hyperpolarized ^13^C MRS	No	Rat model of glutathione depletion (diethyl maleate)	Rate of DHA to Vitamin C conversion decreased by 50% with glutathione depletion
PET	^62^Cu-ATSM	Ikawa et al. (51)	^62^Cu-ATSM PET	Yes	Parkinson disease	Increase in ^62^Cu-ATSM uptake in the bilateral striata in PD compared to HC
		Ikawa et al. (52)	^62^Cu-ATSM PET	Yes	ALS and HC	Increased ^62^Cu-ATSM accumulation in bilateral motor cortices in ALS compared to HC. In those with ALS, SUV correlated with increased disease severity
		Ikawa et al. (53)	^62^Cu-ATSM PET	Yes	MELAS	Cu-ATSM accumulation suggesting oxidative stress within a subacute lesion, decreased within a chronic lesion
		Okazawa et al. (54)	^64^Cu-ATSM PET	Yes	Early Alzheimer disease and HC	Increased Cu-ATSM accumulation in Alzheimer disease compared to HC, particularly in the cingulate cortex and hippocampus
	Superoxide	Hou et al. (55)	^18^F-ROStrace PET	No	Lipopolysaccharide (LPS) mouse model of inflammation	[^18^F]ROStrace rapidly crossed the blood-brain barrier (BBB) of LPS-treated animals but not HC

**Table 2:** Advantages and disadvantages.

**Modality**	**Technique**	**Advantages**	**Disadvantages**
MRI	QUEST MRI	- Uses standard high-field clinical hardware - No exogenous contrast agent - Assesses overall oxidative state as opposed to a single enzyme or antioxidant - High spatial resolution	
	Mito-TEMPO	- Favorable CNS penetration - High spatial resolution - Assesses overall oxidative state	- Rapidly bio-reduced which may limit clinical utility - Exogenous contrast has difficulty crossing the blood-brain barrier - Mito-TEMPO itself is an antioxidant and may alter measurement within the target organ - Not FDA approved for use in human subjects
	Overhauser-enhanced MRI	- Favorable CNS penetration	- Requires specialized equipment platforms that are not broadly available - Requires exogenous contrast agents that are not FDA approved in humans - Exogenous contrast may interact with free radicals in the tissue
	Proton exchange rate (*k*_ex_) MRI	- Does not require exogenous contrast agent - Assesses overall oxidative state	- Long scan times - Single slice scanning for *k*_ex_ mapping - Uses normal appearing white matter as internal reference, which may underestimate *k*_ex_ values
MRS	Glutathione (GSH) MRS	- Robust clinical evidence in humans relative to other modalities - Standard clinical equipment - No exogenous contrast agent - Demonstrated reproducibility (at 7T)	- Techniques not universal across institutions - Single voxel studies limited to evaluation of large voxels due to low cerebral concentrations of GSH - Only assesses one particular antioxidant as opposed to a more comprehensive assessment of oxidative stress pathways
	Vitamin C/dehydroascorbate (DHA) hyperpolarized MRS	- Assesses overall reductive state	- Requires specialized preclinical equipment
PET	^62^Cu-ATSM PET	- May have higher sensitivity than MR-based techniques - Radiotracer blood-brain barrier permeable - Validated image-derived input function allows for quantification without invasive arterial blood sampling	- Mixed biological affinity makes technique sensitive to hypoxia as well as oxidative stress - Low spatial resolution
	^18^F-ROStrace PET	- May have higher sensitivity than MR-based techniques - Radiotracer blood-brain barrier permeable	- Low spatial resolution - May require arterial sampling for quantification

## Magentic resonance imaging

Most conventional MRI methods are insensitive to OS at the cellular level. However, a recently developed technique, QUEnch-assiSTed (QUEST) MRI, may have utility in the identification of OS *in vivo*. As opposed to assessing a single antioxidant or free radical, QUEST-MRI assesses the overall redox state. Free radicals are paramagnetic, and during times of OS they cause shortening of spin-lattice relaxation time (T1) and thus prolongation of the spin-lattice relaxation rate (R1). By assessing a reduction in R1 after a quench with anti-oxidant treatment during the scan (e.g., administration of alpha-lipoic acid) this weights the specific contribution of ROS to the increased R1 (56). However, relaxation rates may be impacted by factors that are not related to ROS—such as water and oxygen content (57, 58). Several QUEST-MRI studies show agreement with free radical measurements assessed *ex vivo* (43, 59). QUEST MRI was also used in a small study of AD-like and Angelman Syndrome mouse model hippocampi which suggested a gradient of excessive free radical production similar to that found in *ex vivo* experiments (42). Whether QUEST-MRI is practical for *in vivo* human studies remains to be seen as studies of this technique to date have all been performed in animal models. Advantages of this technique include the lack of contrast agent and the use of standard clinical MRI hardware. Further assessment of this technique would be of interest in the context of multiple human neurodegenerative diseases.

While quench-assisted MRI techniques utilize an exogenous contrast agent to assess the relationship of ROS production to T1 shortening, the in vivo proton exchange rate (*k*_ex_), aims to be an endogenous contrast-based ROS MRI method capable of assessing oxidative stress. Proton exchange occurs between bulk water and metabolites primarily through endogenous metabolites such as amine, amide, hydroxyl, and hydrosulfuric groups. Using chemical exchange saturation transfer (CEST), protons are excited by a pre-saturation pulse and then magnetization is transferred to the bulk water, which can then be detected by MRI. The resulting proton exchange rate is significantly altered by the presence of ROS and less so by temperature, metabolite concentrations, and pH of the tissue (60). In a study of 16 subjects with RRMS, *k*_ex_ was elevated in gadolinium enhancing lesions, but also in gadolinium-negative, slowly expanding MS lesions which suggests it may be a feasible technique to detect “smoldering” chronically inflamed gadolinium-negative lesions with higher levels of oxidative stress (45). However, this technique may be limited by long scan duration requirements which limits acquisition to single slices.

Another technique utilizes paramagnetic nitroxide contrast agents aimed at detecting ROS. Nitroxides are stable free radicals with a single unpaired electron, capable of shortening the longitudinal relaxation time (T1) and thus providing MRI contrast. Nitroxides have much lower relaxivity compared to conventional gadolinium-based contrast agents, though they do have higher volume of distribution due to improved cell permeability. One drawback is that they are rapidly bio-reduced which has limited their suitability as an MRI-contrast agent (61–63). Mito-TEMPO is a blood-brain barrier- (BBB) and mitochondria-penetrating nitroxide derivative. It has a relatively high contrast on T1 images and specifically scavenges superoxide, accumulating in areas of high superoxide concentration. Studies in MPTP-treated mice, a model of Parkinson's disease, showed high mito-TEMPO concentrations in the dopaminergic areas of the brain—indicating high oxidative activity due to abnormal production of mitochondrial superoxide, concordant with what is found in autopsy studies (44). The ability of mito-TEMPO to penetrate the BBB and mitochondria is advantageous to studying CNS-specific diseases. However, using exogenous MRI redox contrast agents does have limitations. For instance, mito-TEMPO itself acts as an antioxidant that may alter the tissue under evaluation. Additionally, much remains unknown in regards to their pharmacokinetics, safety, and suitability for use in the evaluation of the human brain. Studies of these agents to date has largely been performed in animal models of tumors; Development of these agents for human subjects and neurodegenerative disease is an area of limited evaluation to this point (64).

Overhauser-enhanced MRI (OMRI), builds on electron paramagnetic resonance (EPR) techniques and is another imaging method utilizing nitroxide radicals to assess oxidative stress. Direct EPR has very limited resolution due to the short half-life of nitroxides which are used as contrast agents and is less promising for use *in vivo* in human subjects. To improve the spatial resolution, OMRI indirectly assesses the activity of the nitroxide radical by using the Overhauser effect which transfers large spin polarizations from nitroxide radicals to dipolar-coupled protons, the enhanced spin polriazations of these protons are then imaged to assess free radical ditribution. The adoption of OMRI has been limited due to low sensitivity and the necessity that it is performed at ultra-low magnetic fields. The permeability of TEMPOL across the blood-brain barrier increases in the setting of oxidative stress (65). Dynamic tracking of oxidative stress by monitoring a nitroxide radical antioxidant such as 4-hydroxy-TEMPO (TEMPOL) may be more feasible with new techniques allowing improved speed, sensitivity, and temporal resolution (66). OMRI has been used in *in vivo* studies of animal models of Parkinson disease using the nitroxide radical 3-methoxycarbonyl-2,2,5,5,-tetramethylprrolidine-1-oxyl (methoxy-carbonyl-PROXYL). In this study, OMRI of 6-OHDA-lesioned rats suggested that 6-OHDA induced an environment of oxidative stress and subseuqnet *ex-vivo* spin-trapping demonstrated the direct induction of hydroxyl radical generation associated with mitochondrial dysfunction (46). Although there have been developments in very-low-field MRI systems for human studies, this remains a limitation to this technique at this point.

## Magnetic resonance spectroscopy

Spectroscopy is a powerful, non-invasive method of quantifying metabolites within a region of interest. Magnetic resonance spectroscopic imaging (MRSI) comprises a class of methods, including chemical shift imaging, echo planar spectroscopic imaging, and IDEAL spectral decomposition. MRSI is performed either dynamically, or at a single time-point that is often chosen to coincide with the peak MR signal of the metabolic product of interest. Most MRS studies are based on proton (^1^H) nuclear magnetic resonance (NMR), though other nuclei such as ^13^C or ^31^P can be utilized. MRS offers the advantage of detecting its target in real-time, and it can be performed with standard clinical equipment and without exogenous contrast agents or substantial cost. However, metabolites associated with OS, such as GSH, exist in relatively low concentrations in comparison to other cerebral metabolites, and often large voxels must be examined to account for the low concentration of target molecules, which limits anatomical specificity. Additionally, metabolites with overlapping resonances pose a challenge to resolving separate contributions to spectra, and a reference metabolite, e.g., water, is typically chosen to calculate relative metabolite ratios. Several MRS techniques have been developed to overcome these disadvantages. Likewise, there is a push toward the utilization of universal sequences across institutions and manufacturers which may help minimize technical barriers, develop normative databases, and facilitate inter-institutional collaboration (67).

GSH is one of the best-studied metabolites associated with OS in MS. It is the most abundant antioxidant of the CNS, and the GSH oxidation status (GSH/GSSG) is considered one of the best markers of cellular redox equilibrium (68). Detection of GSH using MRS is technically challenging at clinical field strengths of 1.5 or 3 T due to its low concentration in the human brain coupled with the fact that conventional single-echo acquisitions, typically used for MRS acquisitions, cannot be used to resolve GSH given its overlap with other metabolite resonances. Srinivasan et al. evaluated GSH concentrations in people with MS with single voxel MR spectroscopy at 7T and identified differences in white and gray matter GSH concentrations (47). While they did not identify significant GSH differences in the WM between controls and people with MS, they did observe reduced GSH in MS white matter lesions (47). Since publication of this preliminary and promising investigation, advancement of spectral-edited MRS techniques such as MEGA-PRESS show improved capability and reliability such that assessments of GSH are now possible even at lower field strengths (69–71). Additional work using J-difference edited MRS of GSH at 7T exhibited good reliability in controls and established feasibility in a single MS patient (69).

In more recent studies of GSH in MS, Choi and Lee (49) and Choi et al. (72, 73) used a specially designed selective multiple quantum chemical shift imaging (CSI) technique to assess glutathione concentration throughout a 3 cm thick axial brain slice. GSH concentrations were lower in MS subjects than controls, and lower still in people with progressive MS compared to RRMS (72). Follow-up over a 3–5-year period showed that decreased GSH concentrations persisted in patients with SPMS compared to controls and that participants judged to have clinical worsening had sharper declines in GSH concentrations than clinically stable patients (73). While MRS of glutathione is promising, it is important to note that assessments of GSH reflect only a portion of a tissue's anti-oxidative stress defenses, and that it is not well suited to directly measure the production of excessive ROS from sources such as mitochondria.

Another novel spectroscopic technique uses ^13^C, a nuclear magnetic resonance (NMR)-sensitive isotope of carbon. Thanks to its extensive chemical shift range ^13^C NMR is an attractive method for interrogation of redox states *in vivo* and hyperpolarization (HP) offers a means of improving sensitivity. Although there are many ^13^C metabolic tracers under development, only a few have been evaluated as detectors of redox state. The most studied HP ^13^C-labeled tracers for assessment of redox state *in vivo* are the reduced and oxidized forms of vitamin C: HP[1–^13^C] dehydroascorbic acid (DHA) and [1–^13^C] ascorbic acid (AA). These reflect intracellular and extracellular redox states respectively. The reduction of DHA to AA is mediated by glutathione- or NADPH-dependent mechanisms. Increases in HP[1–^13^C] DHA in tumors appear to reflect increases in glutathione (74). In the cuprizone model of MS, hyperpolarized ^13^C-MRS has been used to characterize inflammatory lesions that attract proinflammatory mononuclear phagocytes. An elevated lactate signal was demonstrated within MS lesions that were histologically confirmed to be rich in mononuclear phagocytes. Moreover, these activated phagocytes had elevated pyruvate dehydrogenase kinase 1 (PDK1), responsible for inhibition of the oxidation of pyruvate by mitochondrial PDH, leading to increased metabolism to lactate in the cytosol. While this technique was aimed at detecting inflammation, there could be overlap in the ability of this technique to detect concomitant OS in areas outside of active lesions (75). Similar techniques have been utilized in only a few in vivo studies of the human brain metabolism and none yet in MS or neurodegenerative disease (76). Barriers to adoption of ^13^C NMR MRS include the need for specialized equipment such as a polarizer, isotope-specific ^13^C head coils, and MRI machines capable of multi-nuclear scans which most clinical machines are not equipped to perform.

## Positron emission tomography

Molecular imaging with positron emission tomography (PET) can directly evaluate subtle biological changes, including redox status. The ^62^Cu-diacetyl-bis(N^4^-methylthiosemicarbazone) (^62^Cu-ATSM) radioligand has been evaluated in neurodegenerative disorders including Mitochondrial Encephalopathy, Lactic Acidosis, and Stroke Like Episodes (MELAS), Parkinson disease, and amyotrophic lateral sclerosis (ALS) (51–53). ^62^Cu-ATSM is a PET radioligand that is a chelate complex containing a radioactive divalent copper. Because of its divalence (Cu^2+^) it is reduced to the monovalent form (Cu^1+^) in tissues with excess electrons, indicating states of increased oxidative stress. This reduced Cu^1+^ dissociates from the ATSM and is retained in the reductive tissues which allows for its detection *via* PET. While it was initially designed to identify areas of hypoxia, it has also been shown to identify tissues with over-reductive states with elevated NADH concentrations in a normoxic state (77). This is believed to be due to the reduced respiratory capacity of local mitochondria.

In regards to studies in other neurodegenerative diseases—^62^Cu-ATSM was evaluated in patients with idiopathic Parkinson's disease which demonstrated increased uptake in the bilateral striata compared to healthy controls, and increased uptake was correlated with increased disease severity (51). Similarly, OS and related mitochondrial dysfunction appears to have a role in the motor neuron degeneration in ALS based on post-mortem studies and the roles of gene mutations (such as SOD1 and TDP-43) which are implicated both in the development of ALS as well as disruption of common oxidative defense pathways. A study using ^62^Cu-ATSM was performed in patients with sporadic ALS—which demonstrated significantly greater accumulation of ^62^Cu-ATSM in the bilateral motor cortices in ALS as compared to healthy controls (52). Another investigation of early Alzheimer disease also suggested increased accumulation of ^62^Cu-ATSM compared to controls (54). As of now this radioligand has not been evaluated in the context of MS, though such investigations may be a useful complement to the MRSI studies described above. Other PET radiotracers are being investigated in the context of OS such as ^18^F-FASu and 18F-ROStrace in other organ systems or targeted for the assessment of neoplasms (78–80). Limited human data is available in neurodegenerative disease using these radioligands and the specificity of these techniques in assessing oxidative stress is an area still under investigation. While PET offers the potential of relatively high sensitivity, it continues to be limited by low spatial resolution relative to MR based techniques.

## Conclusions

OS is a key pathophysiological mechanism underlying neurodegeneration in MS. Because of the complexity of the OS pathways, multiple potential targets to capture OS using *in vivo* imaging techniques have emerged. High spatial resolution methods using MRI offer advantages in the evaluation of MS such as assessing regional or lesional differences in OS. Contrast agents like nitroxides and mito-TEMPO are T1 contrast agents with anti-oxidant properties that penetrate the brain and mitochondria to distribute to tissues where ongoing OS occurs, thus highlighting regional areas with an abundance of ROS. Techniques with high spatial resolution offer the advantage of obtaining more granular regional or lesional variation in OS. A direct measure of overall ROS burden would also be advantageous in the context of MS as opposed to a technique that measures a single ROS, such as glutathione in the case of MRS. In this respect, ^62^Cu-ATSM may offer a sensitive marker that measures OS comprehensively but with a sacrifice in spatial resolution. At present, MRS investigations of glutathione have the most robust literature in the context of MS, but it does have low spatial resolution and requires specific expertise in analysis which has slowed broad uptake of this method. ^62^Cu-ATSM PET appears to be primed for investigations of MS with multiple other studies in neurodegenerative disease, however PET has limitations in spatial resolution that are not ideal. The potential advantages offered by QUEST MRI should encourage further development of this technique for early studies on human subjects with MS. Ultimately performing multimodal studies, for example combining QUEST MRI with susceptibility weighted imaging (SWI) sequences for the detection or iron (81) or PET markers of neuroinflammation (82) may help us obtain multiple layers of information regarding neurodegeneration and the relationship of multiple degenerative processes in MS. Regardless of modality, further investigations of MS using *in vivo* imaging techniques alongside markers of localized neurodegeneration is an important next step in our understanding of the pathophysiology of this still poorly understood aspect of the disease.

## Author contributions

CH and RS proposed the review. CH prepared the tables, and drafted the manuscript for intellectual content. LN, IG, and RB critically reviewed the manuscript with major contributions to intellectual content. All authors read and approved the submitted version.
